# O‐GlcNAcylated TAP1 Impairs Antigen Presentation and Promotes Immune Evasion in Bladder Cancer

**DOI:** 10.1002/advs.202519955

**Published:** 2026-04-24

**Authors:** Jinpeng Wu, Xueting Ren, Zhen Zhai, Lu Wang, Liang Liang, Zengqi Tan, Jiazhen Zhao, Yuxuan Han, Yuhan Li, Feng Guan, Xiang Li

**Affiliations:** ^1^ Key Laboratory of Resource Biology and Biotechnology in Western China Provincial Key Laboratory of Biotechnology College of Life Sciences Ministry of Education Northwest University Xi'an Shaanxi China; ^2^ The Comprehensive Breast Care Center The Second Affiliated Hospital of Xi'an Jiaotong University Xi'an Shaanxi China; ^3^ Department of Urology The First Affiliated Hospital of Xi'an Jiaotong University Xi'an Shaanxi China; ^4^ Shaanxi Province Key Laboratory of Molecular Cardiology School of Medicine Northwest University Xi'an Shaanxi China; ^5^ Institute of Molecular and Translational Medicine (IMTM) and Department of Biochemistry and Molecular Biology Xi'an Jiaotong University Health Science Center Xi'an Shaanxi China

**Keywords:** antigen presentation, bladder cancer, mhc‐I, O‐GlcNAc, tap1

## Abstract

Major histocompatibility complex class I (MHC‐I) proteins play a crucial role in immune surveillance by presenting newly synthesized antigens to CD8^+^ T cells on the cell surface. The downregulation of MHC‐I impairs antigen presentation and facilitates immune evasion in bladder cancer. O‐GlcNAcylation, an O‐linked N‐acetylglucosamine modification that is upregulated in various cancers, is increasingly recognized for its role in immune regulation. However, its specific role in antigen presentation remains unclear. In this study, we identified an inverse correlation between O‐GlcNAcylation levels and immune cell infiltration, specifically reducing CD8^+^ T cell numbers in bladder cancer. Reducing cellular O‐GlcNAcylation significantly enhanced antigen presentation efficiency. We demonstrated that O‐GlcNAcylation of antigen peptide transporter 1 (TAP1), a key transporter in the antigen presentation pathway, disrupts its interaction with MHC‐I, thereby promoting the autophagic‐lysosomal degradation of MHC‐I and impairing CD8^+^ T cell‐mediated cytotoxicity. Inhibiting O‐GlcNAcylation of TAP1 significantly augmented antitumor immune responses. Collectively, these findings reveal a novel mechanism by which O‐GlcNAcylation facilitates immune evasion in bladder cancer and suggest a potential therapeutic strategy for enhancing TAP/MHC‐I‐mediated antigen presentation.

## Graphical abstract

This article unveils a critical mechanism of immune evasion in bladder cancer, which the O‐GlcNAc modification of TAP1 disrupts antigen presentation. This modification triggers HLA‐A degradation, shielding tumor cells from CD8^+^ T cell attack. The findings highlight targeting this pathway as a promising therapeutic avenue to sensitize tumors to immunotherapy.

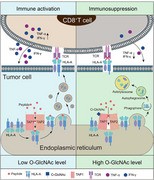

## 1 Introduction

Immune evasion remains a major obstacle in cancer therapy. In bladder cancer, the most prevalent urologic malignancy in males [1, 2], tumors develop sophisticated immune escape mechanisms, including upregulation of immunosuppressive molecules (e.g., PD‐L1) [3], secretion of immunosuppressive cytokines (e.g., TGF‐β, IL‐10) [4], and recruitment of regulatory immune cells (e.g., Tregs, MDSCs) to establish an immunosuppressive niche [5].

A key factor in immune evasion is the presentation of antigens to T cells, a process regulated by the major histocompatibility complex (MHC), which is classified into MHC‐I or MHC‐II [6, 7]. Specifically, classical MHC‐I subtypes (HLA‐A, HLA‐B, and HLA‐C) contain endogenous peptides derived from self‐proteins or intracellular pathogens in CD8^+^ T cells. These peptides are transported to the endoplasmic reticulum (ER) by antigen peptide transporter 1 (TAP1), where they form complexes with MHC I, tapasin, and calnexin. This complex is then trafficked to the cell surface, thereby facilitating peptide presentation for CD8^+^ T cell activation [8, 9]. Downregulation of MHC‐I is observed in up to 80% of human tumors, impairs antigen presentation, and promotes immune evasion [10]. Therefore, strategies to enhance MHC‐I expression represent a promising approach for counteracting tumor immune evasion.

O‐GlcNAcylation (O‐GlcNAc), a critical post‐translational modification catalyzed by the glycosyltransferase OGT and glycosidase OGA, plays a pivotal role in regulating immune homeostasis [11, 12, 13]. Emerging evidence suggests that dysregulated O‐GlcNAc in tumor cells impairs immune checkpoint function and facilitates immune evasion. For instance, elevated O‐GlcNAc in hepatocyte growth factor‐regulated tyrosine kinase substrate (HGS) in tumor cells disrupts its interaction with PD‐L1, thereby inhibiting the PD‐L1 degradation pathway [14]. Conversely, reducing O‐GlcNAc levels enhances natural killer (NK) cell cytotoxicity [15]. However, the precise role of O‐GlcNAc in antigen presentation within tumor cells remains poorly understood.

In this study, we demonstrated that O‐GlcNAc negatively affects MHC‐I expression by promoting autophagic‐lysosomal degradation. Specifically, we found that O‐GlcNAc of TAP1 disrupted its interaction with MHC‐I, thereby suppressing CD8^+^ T cell‐mediated cytotoxicity. Inhibition of TAP1 by O‐GlcNAc increased MHC‐I levels and consequently restored CD8^+^ T cell cytotoxicity both in vitro and in vivo.

## 2 Materials and Methods

### 2.1 Cell Lines and Cell Culture

The normal human bladder mucosal epithelial cell line HCV29 (RRID: CVCL_8228), non‐muscle‐invasive bladder cancer cell line KK47 (RRID: CVCL_8253), and highly aggressive invasive bladder cancer cell line YTS‐1 (RRID: CVCL_6746) were maintained as previously described [16]. Additionally, human uroepithelial cell line SV‐HUC‐1 (RRID: CVCL_3798); transitional carcinoma cell lines T24 (RRID: CVCL_0554) and J82 (RRID: CVCL_0359); human bladder transitional papilloma cell lines RT‐4 (RRID: CVCL_0036) and 5637 (RRID: CVCL_0126); and the mouse bladder carcinoma cell line MB49 (RRID: CVCL_7076) were purchased from the Cell Bank of the Chinese Academy of Sciences (Shanghai, China). YTS‐1, KK47, SV‐HUC‐1, and 5637 cells were cultured in RPMI 1640 medium (Biological Industries, Beit Haemek, Israel) supplemented with 10% fetal bovine serum (FBS) and 1% penicillin/streptomycin. J82 and MB49 cells were cultured in DMEM (HyClone) supplemented with 10% FBS and 1% penicillin/streptomycin. All cell lines were maintained at 37°C in a humidified 5% CO_2_ atmosphere.

### 2.2 CD8^+^ T Cell‐Mediated Tumor Killing Assay

All clinical samples used in this study were approved by the Medical Ethics Committee of Northwest University, China) Approval No. 240826082). Human CD8^+^ T cells were isolated from peripheral blood mononuclear cells (PBMCs), and mouse CD8^+^ T cells were isolated from mice spleens, both using MojoSort Human and Mouse CD8 Nanobeads, respectively (Biolegend, San Diego, CA, USA). The isolated CD8^+^ T cells were cultured in IMDM medium (Thermo Fisher Scientific, Waltham, MA, USA) supplemented with 1000 U/mL IL‐2 (Novoprotein, Suzhou, China). The CD8^+^ T cells were activated by 2 µg/mL PMA and 2.5 µg/mL ionomycin (MedChemExpress, Newark, NJ, USA). For CD8^+^ T cell‐mediated tumor killing, CFSE‐labeled tumor cells were plated overnight and subsequently co‐cultured with activated CD8^+^ T cells for 48 h. Apoptosis was measured using flow cytometry.

### 2.3 Quantitative Real‐Time PCR (qRT‐PCR)

Total RNA was extracted from cells using TRIzol Reagent (CoWin Biotech, Beijing, China), and cDNA was synthesized using a reverse transcription kit (Vazyme Biotech, Nanjing, China). Target gene expression was quantified by real‐time PCR with gene‐specific primers (Table S1). Experiments were performed in triplicate, and gene expression was quantified using the 2^−ΔΔCt^ method [16].

### 2.4 Antibodies

Antibodies against TAP1 (sc‐376796) and Lamp2 (sc‐18822) were purchased from Santa Cruz Biotechnology (Dallas, TX, USA). Antibodies against MHC‐I (A‐8754), HLA‐A (A‐11406), HLA‐B (A‐8752), and HLA‐C (A‐10863) were purchased from Abclonal (Wuhan, China). Antibodies against LC3B (T55992), ATG5 (T56835), p62 (T55546), and Beclin1 (T55092) were obtained from Abmart (Shanghai, China). The antibody against GAPDH (G9545) was obtained from Sigma–Aldrich. Horseradish peroxidase (HRP)‐conjugated goat anti‐mouse IgG (A0216), HRP‐conjugated goat anti‐rabbit IgG (A0208), anti‐FLAG (AF0036), and Alexa Fluor 488‐conjugated goat anti‐rabbit IgG (A0423) were obtained from Beyotime Biotechnology (Nanjing, China).

### 2.5 Lysosomal Immunoprecipitation (IP)

Cells were lysed with a non‐denaturing lysis buffer (10 mm HEPES, 1.5 mm MgCl_2_, and 10 mm KCl) supplemented with a protease inhibitor cocktail for 30 min at 4°C. The cells were then mechanically homogenized using a tissue homogenizer. Cell lysate was then incubated with the anti‐LAMP2 antibody in IP buffer (20 mm HEPES, 150 mm NaCl, and 10 mm KCl) for 2 h at 4°C. Following the addition of 20 µL of resuspended Protein A/G Plus‐Agarose beads (Santa Cruz) and incubation at 4°C, the pellet was washed three times with PBS. Proteins were eluted by resuspending the pellet in 100 µL of loading buffer and boiling for 10 min. After centrifugation, the supernatant was collected for Western blot analysis [17, 18].

### 2.6 BBN‐Induced Urinary Bladder Carcinogenesis in Mice

All animal experiments were approved by the Animal Ethics Committee of the Northwest University (China). Approval No. NWU‐IACUC‐20260105 m. Urinary bladder carcinogenesis was induced in mice, as previously described [19]. Briefly, 6‐week‐old BALB/c mice (Biocytogen Pharmaceuticals, Beijing, China) were fed freshly prepared 0.05% N‐butyl‐N‐(4‐hydroxybutyl)‐nitrosamine (BBN; Aladdin, Shanghai, China) in drinking water for eight weeks. Mice with bladder cancer were randomly treated with OSMI‐1 (1 mg/kg) via tail vein injection every alternate day. After 14 days, the mice were euthanized and bladder tissues were collected for immunohistochemical (IHC) and FACS analyses.

### 2.7 Plasmid and Cloning

Full‐length human TAP1 was amplified by PCR using cDNA from YTS‐1 cells. Site‐directed mutagenesis was performed using fusion PCR. The full‐length TAP1 and site‐directed TAP1 mutants were cloned into the pLVX‐Flag‐hyg plasmid (Addgene, Cambridge, MA, USA). A lentiviral shRNA vector was constructed using the pLKO‐puro plasmid (Addgene, Cambridge, UK). The primer and target sequences for shRNA are listed in Table S2.

### 2.8 Bladder Cancer Xenografts in OT‐1 Mice

A total of 1×10^6^ bladder cancer cells transfected with pLVX‐OVA‐Luc (Addgene) were orthotopically implanted into 4‐week‐old female OT‐1 TCR transgenic mice (Shanghai Model Organisms Center). Tumor progression was longitudinally monitored using weekly bioluminescence imaging (IVIS Spectrum; PerkinElmer). At the end of the study, mice were humanely euthanized for necropsy and subsequent tumor excision, gravimetric analysis, and IHC staining.

### 2.9 In Vitro O‐GlcNAcylation Assay

An in vitro glycosylation assay was performed as described previously [20]. Briefly, 500 ng of His‐tagged TAP1 protein, bound to Ni‐NTA agarose beads, was incubated with 500 ng of OGT and 5 mM UDP‐GlcNAc in a 100 µL reaction buffer (50 mm Tris‐HCl, pH 7.5, 12.5 mm MgCl_2_) for 4 h at 37°C. Following incubation, the beads were washed three times with NP‐40 lysis buffer. The bound proteins were eluted with SDS sample buffer and analyzed by SDS‐PAGE.

### 2.10 Isothermal Titration Calorimetry (ITC)

The ITC assay was performed as previously described [21]. His‐tagged TAP1 protein (100 µm in 250 µL PBS) was loaded into the ITC cell and titrated with either biotin‐labeled peptides or GST‐tagged HLA‐A (1.5 mm in 50 µL PBS) from the syringe. Binding interactions were monitored using a nano‐ITC calorimeter (TA Instruments, Newcastle, MA, USA).

### 2.11 Estimate Score Analysis

The tumor microenvironment (TME) was quantified using the ESTIMATE algorithm (Estimation of STromal and Immune cells in MAlignant Tumor tissues using Expression data) on tumor transcriptome data. Based on single‐sample gene set enrichment analysis (ssGSEA), stromal, immune, and ESTIMATE scores were calculated for each sample by evaluating enrichment levels of predefined stromal and immune cell signature gene sets.

### 2.12 scRNA‐seq Data Analysis

Single‐cell RNA‐seq data were processed using Seurat software (version 4.3; https://satijalab.org/seurat/). Quality control was performed to remove low‐quality cells and technical artifacts. Cells that met the following criteria were retained: 200< nFeature_RNA < 6000, percentage mt < 25%, and nCount_RNA < 25 000. Genes detected in fewer than 100 cells were excluded from the downstream analyses. Data were normalized using the NormalizeData function and scaled using ScaleData. The top 2000 highly variable genes (HVGs) were identified using FindVariable features. Batch effects across samples were corrected using harmony, with the origin specified as a batch variable. The top 20 Harmony‐corrected principal components were used for downstream analyses, including UMAP visualization, nearest‐neighbor graph construction, and graph‐based clustering. Cell clustering was performed at a resolution of 0.5.

### 2.13 Statistical Analysis

Data are presented as mean ±SD from three independent experiments unless otherwise specified. A two‐tailed Student's t‐test was used for comparisons between two groups, and differences were considered statistically significant at *p* <0.05. ^*^, *p* <0.05; ^**^, *p* <0.01; ^***^, *p* <0.001.

## 3 Results

### 3.1 Dysregulated O‐GlcNAc Is Involved in Bladder Cancer Immune Evasion

**FIGURE 1:**
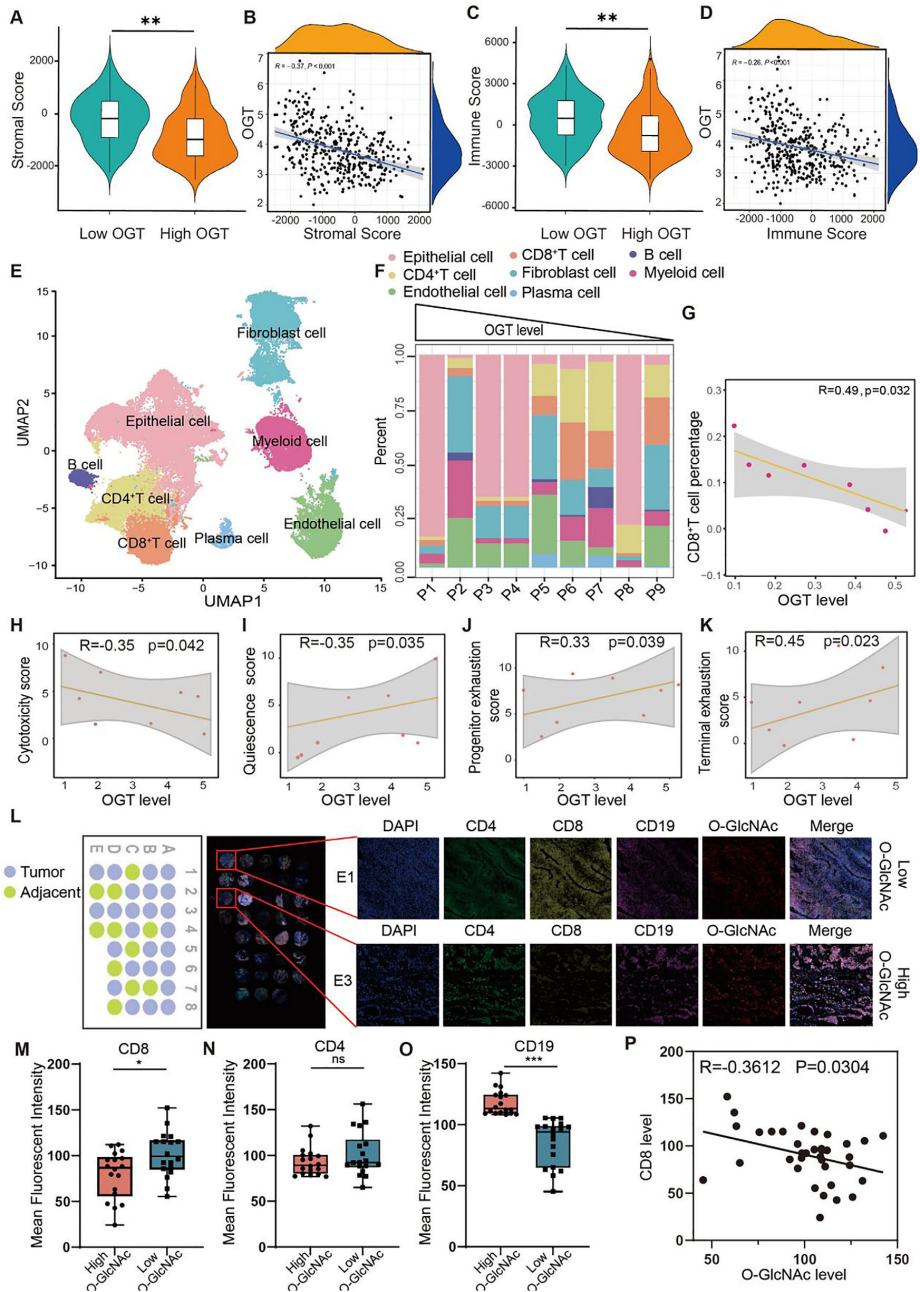
OGT expression correlates with tumor immunity in bladder cancer. (A,B). Correlation between OGT mRNA and stromal scores of bladder cancer patients (low OGT, *n* = 124; high OGT, *n* = 125). (C,D). Correlation of OGT expression with immune score. (E). UMAP plot showing cell clusters by type in pooled single‐cell data from 9 bladder cancer patients. (F). Correlation between OGT mRNA levels and cell type proportions. (G). The correlation between OGT mRNA and CD8^+^ T cell percentage. (H–K). Correlation of OGT levels with CD8^+^ T cell (H) cytotoxicity, (I) quiescence, (J) progenitor exhaustion, and (K) terminal exhaustion. L. Immunofluorescence staining of immune markers in TMA. Representative images show the infiltration of CD19^+^ B cells, CD8^+^ T cells, and CD4^+^ T cells. (M–O). The statistical analysis of CD8^+^ T cell (M), CD4^+^ T cell (N), and CD19^+^ B cell (O) marker expression in TMA (high O‐GlcNAc, *n* = 18; low O‐GlcNAc, *n* = 18). P. Correlation between O‐GlcNAc level and CD8 expression level in TMA (*n* = 36).

Given the involvement of O‐GlcNAc in immune regulation and evasion [11, 14], we investigated OGT expression in the bladder TME. OGT expression levels were negatively correlated with both stromal and immune scores in bladder cancer tissues, but positively associated with tumor purity, indicating a higher proportion of tumor cells within the microenvironment (Figure 1A‐D; Figure S1A,B). Analysis of single‐cell RNA sequencing data (GSE222315) from 9 bladder cancer revealed that higher OGT expression was associated with a reduced proportion of CD8^+^ T cells. Conversely, the proportions of plasma and epithelial cells were positively correlated with OGT levels (Figure 1E,F; Figure S1C,D). Notably, the decrease in the proportion of CD8^+^ T cells was particularly pronounced with increasing OGT expression (Figure 1G; Figure S1E). Elevated OGT levels also diminished CD8^+^ T cell‐mediated cytotoxicity and increased the quiescent and exhausted states of these cells (Figure 1H–K). Multiplex immunofluorescence staining of tissue microarrays (TMAs) revealed that high O‐GlcNAc levels in bladder cancer samples were positively correlated with a reduction in CD8^+^ T cells, consistent with the single‐cell analysis results (Figure 1L–P). These findings suggest that O‐GlcNAc promotes tumor immune evasion by impairing CD8^+^ T cell responses in bladder cancer.

### 3.2 Inhibition of OGT Enhances T Cell‐Dependent Tumor Suppression

**FIGURE 2:**
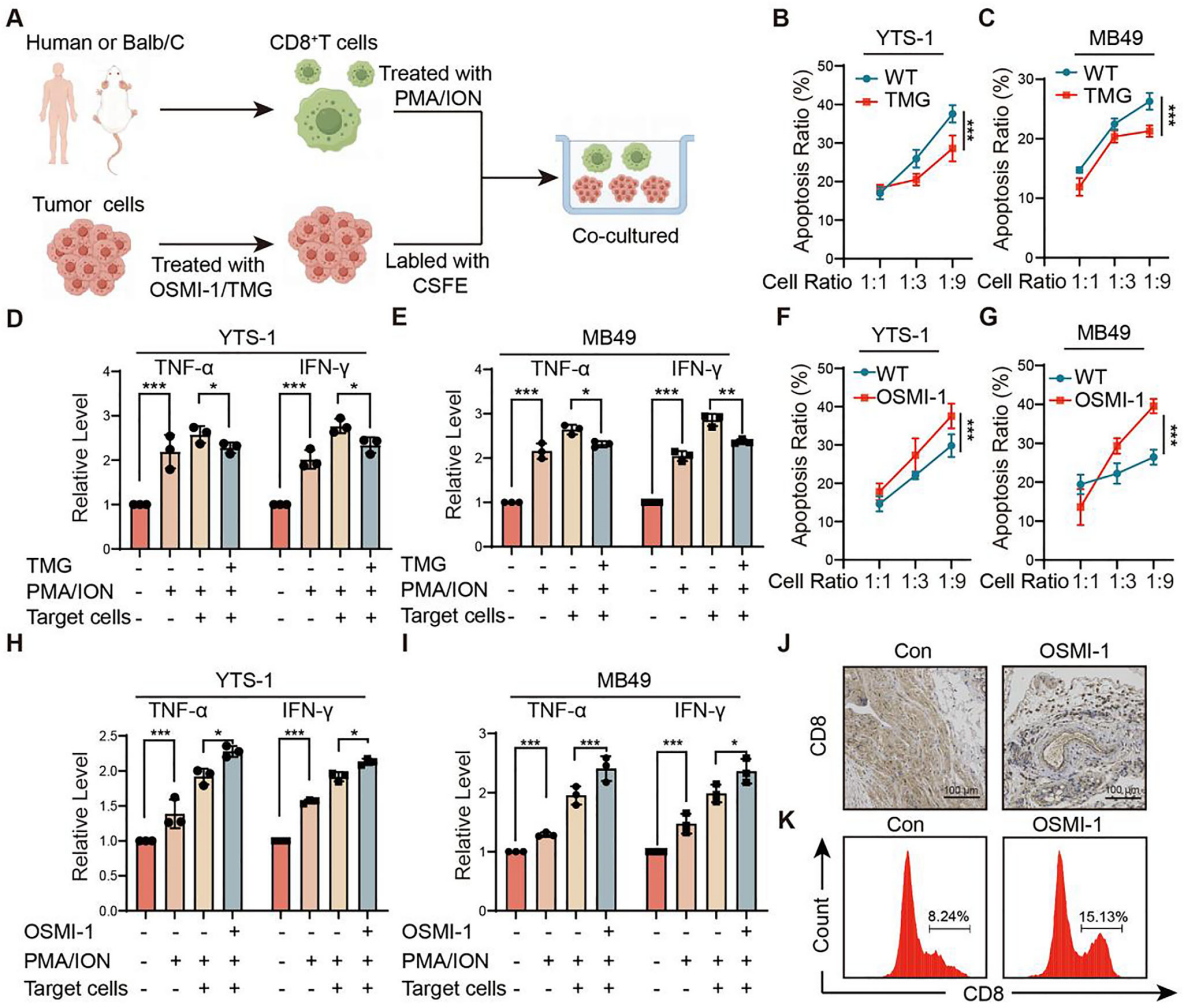
The influence of O‐GlcNAc on T cell‐dependent tumor suppression. (A). Schematic for CD8^+^ T cell‐mediated killing assays. (B,C). CD8^+^ T cell‐mediated cytotoxicity against YTS‐1 cells (B) or BM49 cells (C) after treatment with 20 µm TMG for 48 h. (D,E). Relative levels of TNF‐α and IFN‐γ in the CD8^+^ T cells co‐cultured with TMG‐treated YTS‐1(D) or BM49 (E). (F,G). CD8^+^ T cell‐mediated cytotoxicity against YTS‐1 (F) or BM49 (G) after treatment with 40 µg/mL OSMI‐1 for 48 h. (H,I). Relative levels of TNF‐α and IFN‐γ in CD8^+^ T cells co‐cultured with OSMI‐1‐treated YTS‐1 (H) or BM49 (I). (J). IHC staining of CD8 in paraffin sections of murine bladder cancer tissue. (K). Tumor‐infiltrating CD8^+^ T cells of the murine bladder cancer model detected by flow cytometry.

To elucidate the role of O‐GlcNAc in immune evasion of bladder cancer cells, we performed T cell‐mediated cytotoxicity assays against bladder tumor cells (Figure 2A; Figure S2A). Pretreatment of human (YTS‐1) and mouse (MB49) bladder cancer cells with the OGA inhibitor, Thiamet G (TMG), attenuated CD8^+^ T cell‐mediated cytotoxicity (Figure 2B,C; Figure S2B,C) and suppressed IFN‐γ and TNF‐α secretion (Figure 2D,E). Conversely, inhibition of OGT with OSMI‐1 enhanced CD8^+^ T cell‐mediated cytotoxicity and increased IFN‐γ and TNF‐α production (Figure 2F–I; Figure S2D,E). Similar results were observed in KK47 and T24 cells (Figure S2F–K). In BBN‐induced murine bladder cancer model (Figure S2L–O), we further confirmed that OSMI‐1 treatment increased CD8^+^ T cell infiltration into bladder tumor tissues (Figure 2J,K). Collectively, these results suggest that reducing O‐GlcNAc levels in tumor cells can suppress tumor growth by enhancing CD8^+^ T cell activity.

#### 3.2.1 MHC‐I Expression in Bladder Cancer

**FIGURE 3:**
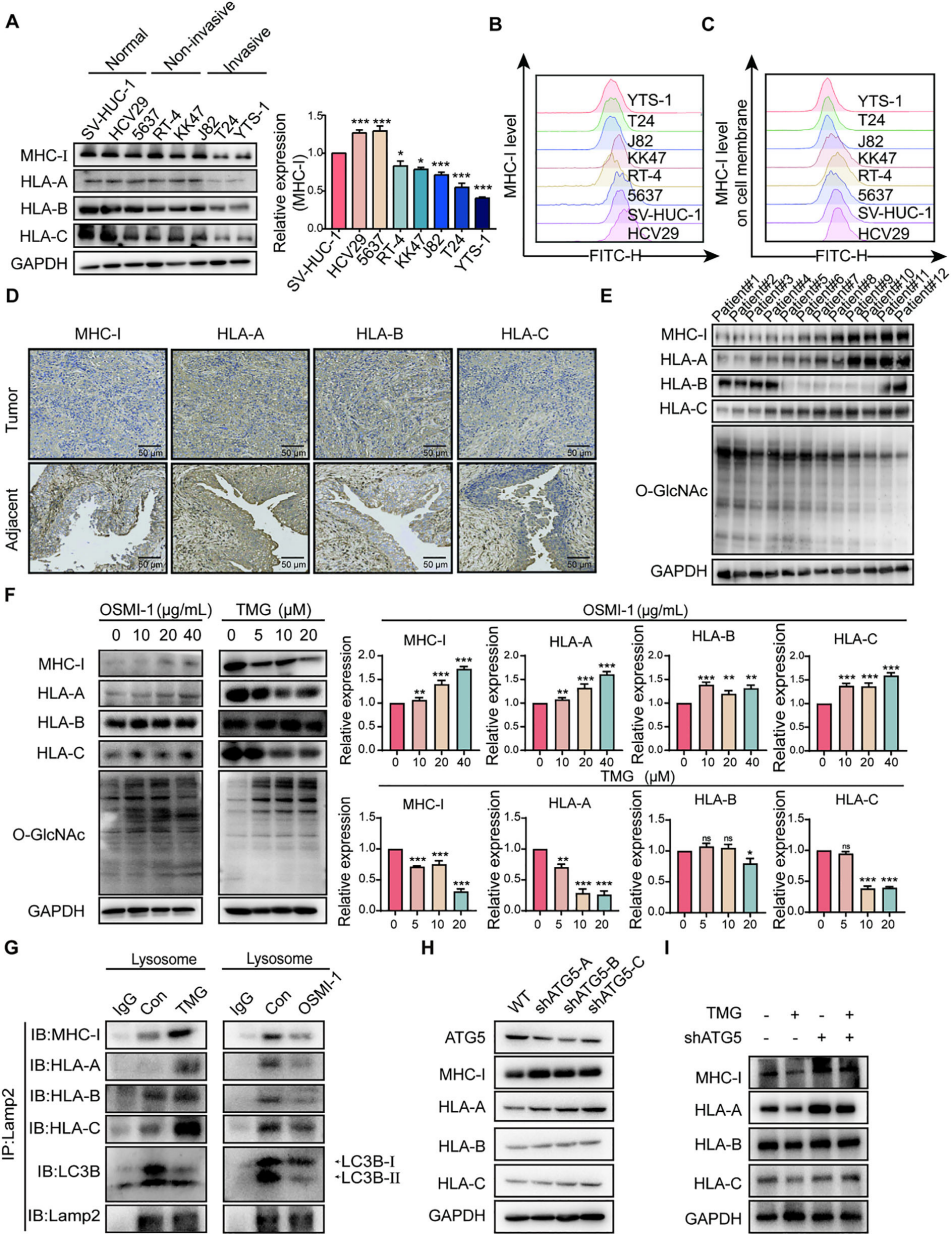
The correlation between O‐GlcNAc and MHC‐I class. (A). Expression of MHC‐I, HLA‐A, HLA‐B, and HLA‐C in different cell lines was detected by Western blot. (B,C). Expression of the total (B) or membranous (C) MHC‐I was detected by flow cytometry. (D). Representative IHC images of MHC‐I, HLA‐A, HLA‐B, and HLA‐C in tumor and adjacent tissues. (E). Expression of MHC‐I, HLA‐A, HLA‐B, and HLA‐C in tumor tissues from bladder cancer patients (*n* = 12). (F). Expression of MHC‐I, HLA‐A, HLA‐B, HLA‐C, and O‐GlcNAc in YTS‐1 cells treated with OSMI‐1 or TMG. (G). Levels of MHC‐I, HLA‐A, HLA‐B, HLA‐C, and LC3B in lysosomes were detected in TMG or OSMI‐1 treated YTS‐1 cells using lysosome immunoprecipitation. (H). Effect of *ATG5* knockdown on MHC‐I molecules in YTS‐1 cells. (I). Effect of TMG treatment on MHC‐I molecules in *ATG5* knockdown cells.

Next, we investigated how O‐GlcNAc influenced the cytotoxicity of CD8^+^ T cells by examining the protein expression of MHC‐I and its subtypes (HLA‐A, HLA‐B, and HLA‐C), which are crucial for presenting endogenous antigens on the cell surface [22]. MHC‐I expression was highest in normal bladder epithelial cells (HCV29 and SV‐HUC‐1), lower in non‐invasive bladder cancer cells (5637, RT‐4, and KK47), and lowest in invasive bladder cancer cells (YTS‐1, T24, and J82) (Figure 3A–C). Consistent with these findings, MHC‐I, HLA‐A, HLA‐B, and HLA‐C levels were significantly downregulated in bladder cancer tissues compared to adjacent epithelial tissues (Figure 3D; Figure S3A). Analysis of bladder cancer samples revealed an inverse correlation between O‐GlcNAc levels and the expression of MHC‐I, HLA‐A, and HLA‐C (Figure 3E; Figure S3B). Specifically, MHC‐I, HLA‐A, and HLA‐C (but not HLA‐B) expression increased following OSMI‐1 treatment or OGT silencing (Figure 3F; Figure S3C). Conversely, TMG treatment or OGT overexpression reduced MHC‐I, HLA‐A, and HLA‐C expression (Figure 3F; Figure S3D). This inhibitory effect of OGT on MHC‐I expression was further validated by flow cytometry (Figure S3E). In the BBN‐induced murine bladder cancer model, we also confirmed that OSMI‐1 treatment increased MHC‐I expression in bladder tumor tissues (Figure S3F). Notably, the mRNA levels of HLA‐A, HLA‐B, and HLA‐C in YTS‐1 cells remained unaffected by either OSMI‐1 or TMG treatment (Figure S3G), suggesting that O‐GlcNAc regulated MHC‐I stability is regulated at the post‐translational level via O‐GlcNAc.

MHC‐I, HLA‐A, and HLA‐C were enriched in lysosomes following TMG treatment of YTS‐1 cells, whereas OSMI‐1 treatment led to decreased lysosomal localization (Figure 3G). Using MG132 (a proteasome inhibitor) and chloroquine (Chl, a lysosomal inhibitor), we confirmed that MHC‐I molecules were primarily degraded via the lysosomal pathway (Figure S3H). Building on existing evidence of autophagy‐mediated MHC‐I degradation, we further examined autophagic activity [17]. Our data demonstrated a significant increase in the LC3B‐II/LC3B‐I ratio (a key indicator of autophagy) in TMG‐treated YTS‐1 cells, whereas OSMI‐1 treatment decreased this ratio (Figure 3G). *ATG5* knockdown, which is essential for autophagosome formation, suppressed autophagy and consequently increased MHC‐I protein levels without affecting mRNA levels (Figure 3H; Figure S3I) [23]. Notably, the TMG‐induced decrease in MHC‐I was abolished in *ATG5*‐deficient YTS‐1 cells (Figure 3I), directly demonstrating that O‐GlcNAc promotes autophagy‐mediated degradation of MHC‐I in bladder cancer.

### 3.3 Inhibition of O‐GlcNAc Enhances HLA‐A/TAP1 Interaction

**FIGURE 4:**
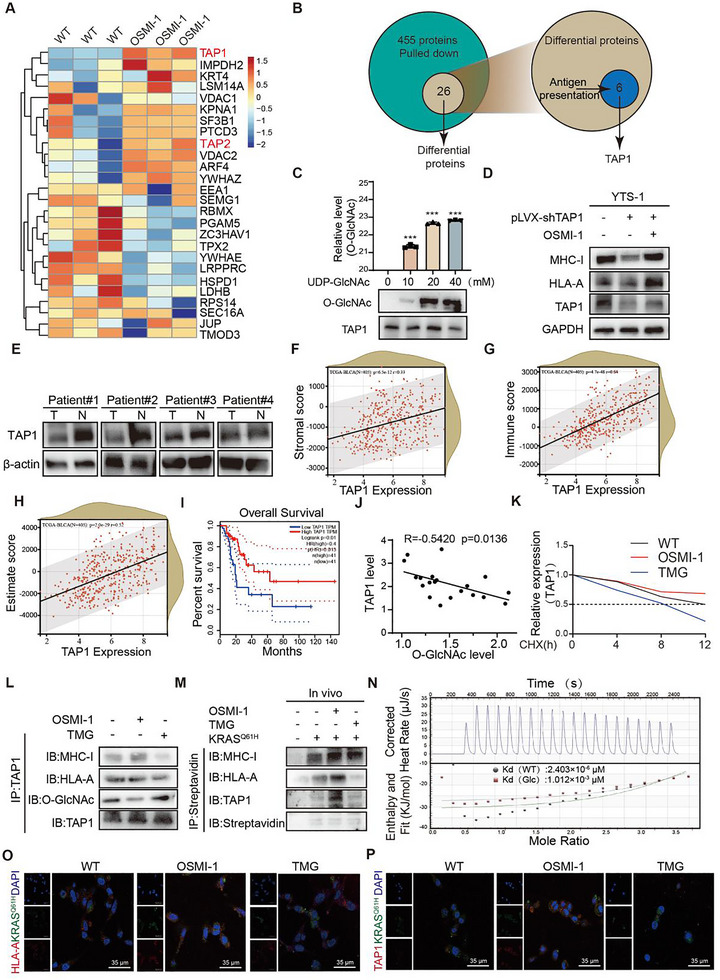
The influence of O‐GlcNAc on the interaction between HLA‐A and TAP1. (A). Heatmap of differentially interacting proteins with HLA‐A in OSMI‐1 treated and non‐treated YTS‐1 cells. (B). Schematic diagram for screening TAP1. (C) TAP1 modified with O‐GlcNAc in vitro and detected by western blot. (D). Effect of OSMI‐1 treatment on MHC‐I and HLA‐A levels in TAP1 knockdown cells. (E). Expression of TAP1 in tumor and adjacent tissues from bladder cancer patients. (F–H). Correlation between TAP1 mRNA and stromal scores (4F), immune score (4G), and estimate score (4H) of bladder cancer patients (low TAP1, n = 203; high TAP1, n = 202). (I). Kaplan‐Meier analyses of the overall survival of bladder cancer patients with low versus high expression of TAP1 (low TAP1, n = 41; high TAP1, n = 41). (J). The Pearson correlation between O‐GlcNAc and TAP1 in bladder cancer tissues (n = 20). (K). Expression of TAP1 in YTS‐1 cells treated with OSMI‐1 or TMG cells treated with CHX at various time points. (L). Interactions of TAP1 with MHC‐I and HLA‐A, and O‐GlcNAc on TAP1 in YTS‐1 cells treated with OSMI‐1 or TMG were detected by Co‐IP. (M). Biotin‐labeled KRASQ61H was incubated with YTS‐1 cells. The interaction of biotin‐labeled KRASQ61H with MHC‐I, HLA‐A, and TAP1 was detected by tricine SDS‐PAGE and western blot. (N). Affinity between biotin‐labeled KRASQ61H and O‐GlcNAc‐modified vs. unmodified TAP1 was detected by ITC. (O,P). Co‐localization of HLA‐A/KRASQ61H (O) and TAP1/ KRASQ61H (P) in YTS‐1 cells treated with OSMI‐1 or TMG.

To elucidate how O‐GlcNAc regulates MHC class I antigen presentation, we performed immunoprecipitation‐mass spectrometry (IP‐MS) to analyze HLA‐A‐interacting proteins in OSMI‐1‐treated and untreated YTS‐1 cells. We identified 26 differentially associated proteins (13 upregulated and 13 downregulated) (Figure 4A; Figure S4A,B) that were functionally enriched in antigen presentation pathways (Figure S4C,D). Interestingly, TAP1 exhibited the most significant alterations in HLA‐A binding and pathway association (Figure 4B, Figure S4E), and was O‐GlcNAcylated (Figure 4C; Figure S4F). Although O‐GlcNAc levels correlated with HLA‐A expression, the absence of detectable O‐GlcNAc in HLA‐A suggested indirect regulation (Figure S4G,H). TAP1 knockdown compromised HLA‐A expression (Figure 4D), indicating that HLA‐A stability is regulated by TAP1. Accordingly, OSMI‐1 expression increased, whereas the expression of TMG and TAP1 decreased (Figure S4I). TAP1 expression was significantly reduced in bladder tumor tissues (Figure 4E). When TAP1 expression is reduced or its function is impaired, tumor antigen peptides cannot be effectively transported into the ER. This leads to insufficient peptides for MHC‐I molecules, consequently preventing their stable presentation on the cell surface, thereby mediating immune escape. Furthermore, TAP1 levels were positively correlated with stromal and immune cell scores; higher TAP1 levels were associated with increased infiltration of immune and stromal cells into the TME (Figure 4F–H). Moreover, patients with high TAP1 expression exhibited better survival curves (Figure 4I). These findings indicate its potential as a biomarker and therapeutic target for cancer diagnosis, prognostic assessment, and immunotherapy. ELISA of 20 bladder cancer tissue samples revealed a negative correlation between O‐GlcNAc and TAP1 levels (Figure 4J). Treatment of YTS‐1 cells with OSMI‐1 significantly increased the half‐life of TAP1, whereas TMG treatment significantly decreased it (Figure 4K; Figure S4J). Additionally, TMG treatment disrupted the TAP1/HLA‐A interaction (Figure 4L). OSMI‐1 treatment enhanced binding of the KRAS^Q61H^ peptide (ILDTAGHEEY) to the TAP1/HLA‐A complex in YTS‐1 cells, whereas TMG treatment markedly reduced this interaction (Figure 4M) [24]. ITC analysis demonstrated that O‐GlcNAcylated TAP1 exhibited reduced KRAS^Q61H^ peptide‐binding affinity (Figure 4N). Consistent with these findings, immunofluorescence revealed enhanced antigen presentation capacity in OSMI‐1‐treated cells, but diminished capacity in TMG‐treated cells (Figure 4O,P). Collectively, these results demonstrate that O‐GlcNAc on TAP1 inhibits the antigen presenting capacity of the TAP1/HLA‐A complex.

### 3.4 Impact of O‐GlcNAc on TAP1 and Antigen Presentation

**FIGURE 5:**
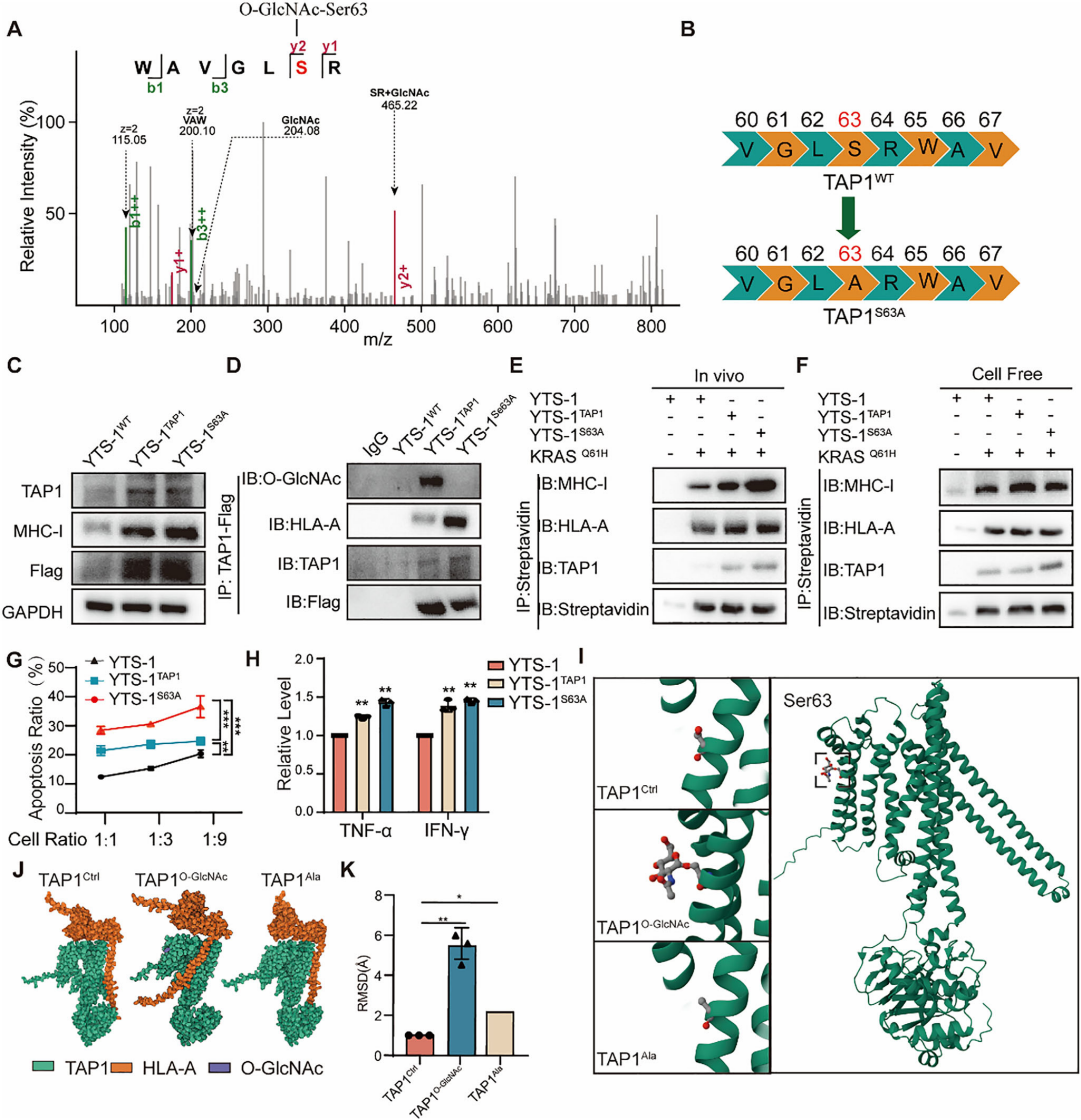
The influence of O‐GlcNAc at Ser63 of TAP1 on antigen presentation. (A). The sites of TAP1 O‐GlcNAcylation were mapped using mass spectrometry. (B).The schematic of the TAP1 mutant at the O‐GlcNAcylation site. (C). Expression of TAP1, MHC‐I, and Flag in YTS‐1 cells expressing TAP1 and TAP1^S63A^. (D). TAP1/HLA‐A, TAP1/O‐GlcNAc interactions in YTS‐1, YTS‐1^TAP1^, and YTS‐1^S63A^ cells, assayed by IP and western blot. (E). Biotin‐labeled KRAS^Q61H^ was incubated with YTS‐1, YTS‐1^TAP1^, and YTS‐1^S63A^ cells. Then, the interaction of KRAS^Q61H^/MHC‐I, KRAS^Q61H^/HLA‐A, and KRAS^Q61H^/TAP1 was detected by tricine SDS‐PAGE. (F). Cell lysates of YTS‐1, YTS‐1^TAP1^, and YTS‐1^S63A^ were incubated with biotin‐labeled KRAS^Q61H^ at 37°C for 24 h. The interaction of KRAS^Q61H^/MHC‐I, KRAS^Q61H^/HLA‐A, and KRAS^Q61H^/TAP1 was detected by tricine SDS‐PAGE. (G). CD8^+^ T cell‐mediated cytotoxicity against YTS‐1, YTS‐1^TAP1^, and YTS‐1^S63A^ cells. (H). Concentration of TNF‐α and IFN‐γ in CD8^+^ T cells co‐cultured system with various TAP1 mutant YTS‐1 cells. (I). Initial structures of TAP1 with the O‐GlcNAcylation and mutation sites labeled and colored by atom types, respectively. (J). Snapshots of TAP1/HLA‐A complexes in TAP, TAP1^S63A^, and TAP1^O‐GlcNAc^ models. (K). The root means square deviation (RMSD) values of TAP1/HLA‐A complexes in TAP1, TAP1^S63A^, and TAP1^O‐GlcNAc^ models.

To investigate the role of O‐GlcNAc in TAP1, the specific O‐GlcNAc site of Ser63 was identified using tandem mass spectrometry (MS/MS) (Figure 5A). We generated YTS‐1^S63A^ cells expressing the TAP1‐S63A mutant [25] (Figure 5B). The mutation at the O‐GlcNAc site at Ser63 of TAP1 resulted in a significant increase in the half‐life of the protein (Figure S5A,B). Elevated MHC‐I and TAP1 expression was observed in both YTS‐1^TAP1^ and YTS‐1^S63A^ cells. Notably, HLA‐A upregulation was more pronounced in YTS‐1^S63A^ cells (Figure 5C). YTS‐1^S63A^ cells demonstrated reduced O‐GlcNAc on TAP1 while simultaneously exhibiting enhanced HLA‐A/TAP1 binding (Figure 5D) and stronger antigen presentation than YTS‐1^TAP1^ cells (Figure 5,5F; Figure S5C). A marked reduction in lysosomal accumulation of MHC‐I and HLA‐A was observed in YTS‐1^TAP1^ cells, and this reduction was particularly pronounced in the YTS‐1^S63A^ mutant (Figure S5D). In a co‐culture system, CD8^+^ T cells displayed enhanced killing of YTS‐1^S63A^ cells compared to YTS‐1^TAP1^ cells, and also showed increased secretion of TNF‐α/IFN‐γ (Figure 5G,H; Figure S5E). Molecular dynamics simulations revealed that neither O‐GlcNAc on Ser63 nor its mutation to Ala significantly affected TAP1 backbone stability (Figure 5I; Figure S5F). However, RMSD analysis demonstrated structural perturbations and conformational reorganization within the TAP1^O‐GlcNAc^/HLA‐A complex (Figure 5J,K). To further investigate whether this structural perturbation was directly caused by O‐GlcNAc, we expressed and purified non‐O‐GlcNAcylated TAP1 and HLA‐A in *E. coli*, followed by in vitro glycosylation of TAP1. Using ITC, we examined the interaction between HLA‐A and TAP1^Ctrl^ as well as TAP1^O‐GlcNAc^ and revealed a significantly reduced interaction between HLA‐A and TAP1^O‐GlcNAc^ (Figure S5G,H).

### 3.5 Inhibition of O‐GlcNAc on TAP1 Reduces Immune Evasion in Bladder Cancer

**FIGURE 6:**
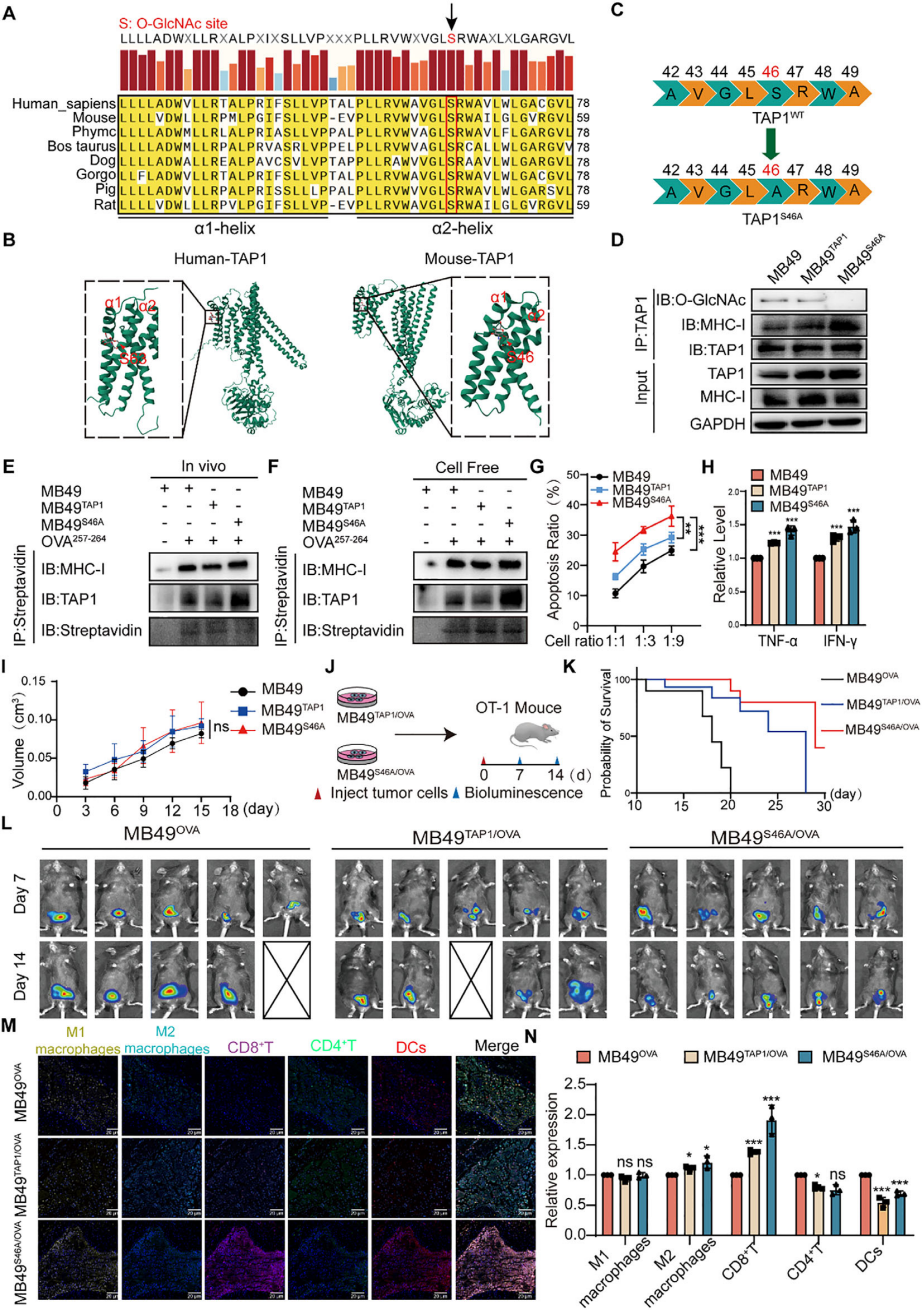
The influence of O‐GlcNAcylation of TAP1 on immune escape. (A). Homology analysis of TAP1 in various species. (B). TAP1‐TAP2‐MHC‐I complex snapshots of mouse or human. (C). The schematic of the mouse TAP1 O‐GlcNAcylation mutant. (D).TAP1/MHC‐I and TAP1/O‐GlcNAc interactions were detected in MB49, MB49^TAP1^, and MB49^S46A^ assayed by IP and western blot. (E). Biotin‐labeled OVA^257‐264^ was incubated with MB49, MB49^TAP1^, and MB49^S46A^ cells. Then, the interaction of OVA^257‐264^ with MHC‐I, HLA‐A and TAP1 was detected by tricine SDS‐PAGE and western blot. (F). The interaction of biotin‐labeled OVA^257‐264^ with MHC‐I, HLA‐A, and TAP1 were assay in cell free system. (G). CD8^+^ T cell‐mediated cytotoxicity against MB49, MB49^TAP1^, and MB49^S46A^ cells. (H). Relative levels of TNF‐α and IFN‐γ in the CD8^+^ T cells co‐cultured with various MB49 cells. (I). Volumes of tumors from *BALB/c* nude mice injected with MB49, MB49^TAP1^, and MB49^S46A^ cells. (J). The schematic of CD8^+^ T cell‐mediated tumor cytotoxicity in vivo. (K). Survival curves of mice that were injected with MB49^OVA^, MB49^TAP1/OVA^, and MB49^S46A/OVA^ cells (*n* = 5). (L). Tumors formed by MB49^OVA^, MB49^TAP1/OVA^, and MB49^S46A/OVA^ cells were detected by live animal bioluminescence imaging. (M). Immunofluorescence staining of immune markers in tumor tissues. Representative images show the infiltration of CD86^+^ M1 macrophages, CD206^+^ M2 macrophages, CD8^+^ T cells, CD4^+^ T cells, and CD11c^+^ dendritic cells. (N). The statistical analysis of immune cell marker expression in tumor.

The α2‐helix (amino acids 53–78 in human) of TAP1 is highly conserved across multiple species (Figure 6A). In murine TAP1, Ser46 is structurally equivalent to human Ser63 (Figure 6B). We subsequently generated two MB49‐derived cell lines, MB49^TAP1^, transduced with a TAP1‐overexpression lentiviral vector, and MB49^S46A^, transduced with a lentiviral vector encoding a TAP1 S46A mutant (in which Ser46 was replaced by alanine) (Figure 6C). The O‐GlcNAc level in TAP1 decreased in MB49^S46A^ cells, which also exhibited an enhanced interaction between MHC‐I and TAP1 (Figure 6D). In vivo, MB49^S46A^ cells exhibited increased OVA^257‐264^ binding to TAP1 and MHC‐I (Figure 6E,F). A CD8^+^ T cell‐mediated tumor‐killing assay revealed increased cytotoxicity of CD8^+^ T cells against MB49^S46A^ cells compared to MB49^TAP1^ or MB49 cells (Figure 6G). CD8^+^ T cells co‐cultured with MB49^S46A^ cells secreted higher levels of TNF‐α and IFN‐γ than those co‐cultured with MB49^TAP1^ cells (Figure 6H). Xenograft mouse experiments showed no statistically significant differences in tumor volume or growth kinetics among mice receiving MB49, MB49^TAP1^, or MB49^S46A^ inoculation (Figure 6I; Figure S6A,B). These results indicated that while TAP1 overexpression or disruption of its O‐GlcNAc site did not affect tumor growth capacity, it did influence cell sensitivity to CD8^+^ T cells.

We further transduced an OVA‐Luc reporter into MB49 cells and orthotopically injected them into the bladders of OT‐1 mice (Figure 6J), which harbor OVA‐specific CD8^+^ T cells [26]. Compared to OT‐1 mice injected with MB49^OVA^ and MB49^TAP1/OVA^ cells, those injected with MB49^S46A/OVA^ cells displayed reduced volumetric expansion and significantly prolonged survival (Figure 6K,L). The infiltration of dendritic cells (DCs) was significantly reduced, whereas the frequencies of M2 macrophages and CD8^+^ T cells were increased in MB49^S46A/OVA^ tumors. Notably, CD8^+^ T cell infiltration exhibited the most pronounced increase among the immune populations (Figure 6M,N; Figure S6C). These results demonstrate that O‐GlcNAc on TAP1 is critical for facilitating immune evasion in bladder cancer and that inhibiting this modification enhances antigen presentation and boosts CD8^+^ T cell‐mediated anti‐tumor responses to prevent immune escape.

## 4 Discussion

Immune evasion is a central mechanism contributing to cancer initiation, progression, and therapeutic resistance. Tumor cells contribute to immune evasion by suppressing the recruitment and activity of immune cells, including CD8^+^ T cells and M1 macrophages, thereby fostering a profoundly immunosuppressive TME. The expression and function of numerous immunoregulatory molecules can be modulated by posttranslational modifications, including neddylation, phosphorylation, and various forms of glycosylation [27, 28, 29]. O‐GlcNAc, a unique form of glycosylation, plays a crucial role in maintaining immune homeostasis [11]. For instance, elevated O‐GlcNAc in HGS disrupts lysosomal degradation of PD‐L1, thereby promoting immune evasion [14]. Our previous study demonstrated that bladder cancer exhibits elevated glucose uptake and metabolic flux toward UDP‐GlcNAc, ultimately leading to enhanced protein O‐GlcNAc [30]. Building upon this, the present study demonstrated an association between elevated O‐GlcNAc levels and impaired CD8^+^ T cell responses in bladder cancer.

Elevated O‐GlcNAc levels in CD8^+^ T cells induce significant metabolic reprogramming, shifting the cells from oxidative phosphorylation in the resting state to rapid aerobic glycolysis, which supports their proliferation and effector functions [31, 32]. Based on these findings, the elevated levels of O‐GlcNAc within the TME are expected to enhance the function and infiltration of CD8^+^ T cells. Contrary to this expectation, our study demonstrated that elevated levels of OGT mRNA and overall O‐GlcNAc in the TME were associated with a reduction in the proportion of infiltrating immune cells, particularly CD8^+^ T cells. This discrepancy suggests that changes in O‐GlcNAc levels within CD8^+^ T cells may not be the primary determinant of immune cell infiltration or overall immune homeostasis within the TME. Therefore, our investigation primarily focused on the effect of O‐GlcNAc modulation in tumor cells.

An effective antitumor immune response typically requires key steps, including the presentation of tumor‐associated antigens (TAAs) by the TAP1/HLA‐A complex, is essential for initiating CD8^+^ T cell‐mediated immunity [33, 34]. In this study, we report that MHC‐I expression is regulated by O‐GlcNAc. Interestingly, while no O‐GlcNAc modification was detected on MHC‐I itself, a significant O‐GlcNAc modification was identified on TAP1 (a key component that forms a complex with MHC‐I during antigen presentation) using a glycoproteomics approach. TAP1 mediates the transport of antigenic peptides from the cytoplasm to the endoplasmic reticulum lumen for loading onto MHC‐I cells [35]. We found that O‐GlcNAc in TAP1 disrupted the assembly of the TAP/HLA‐A antigen presentation complex. Consequently, unassembled HLA‐A is targeted for degradation via the autophagic‐lysosomal pathway, leading to reduced MHC‐I surface expression and impaired T cell cytotoxicity, consistent with previously reported autophagic‐lysosomal degradation of MHC‐I in pancreatic cancer, which promotes immune evasion [17]. Downregulation or functional impairment of the TAP/HLA‐A complex is a common immune evasion strategy in multiple cancers [34, 36]. Thus, strategies aimed at reducing O‐GlcNAc levels in bladder cancer cells to enhance TAP/HLA‐A‐mediated antigen presentation, in conjunction with monoclonal antibody therapy or adoptive T‐cell transfer, may represent a promising therapeutic strategy.

O‐GlcNAc at Ser63, located in the N‐terminal region of TAP1, is not directly involved in antigen binding. Instead, it may alter the conformational dynamics of the TAP1/TAP2 peptide‐binding chamber, thereby influencing the transport efficiency of tumor‐associated antigens. Future studies should validate this mechanistic model using cryo‐EM and in situ crosslinking mass spectrometry to provide structural and functional insights.

**FIGURE 7:**
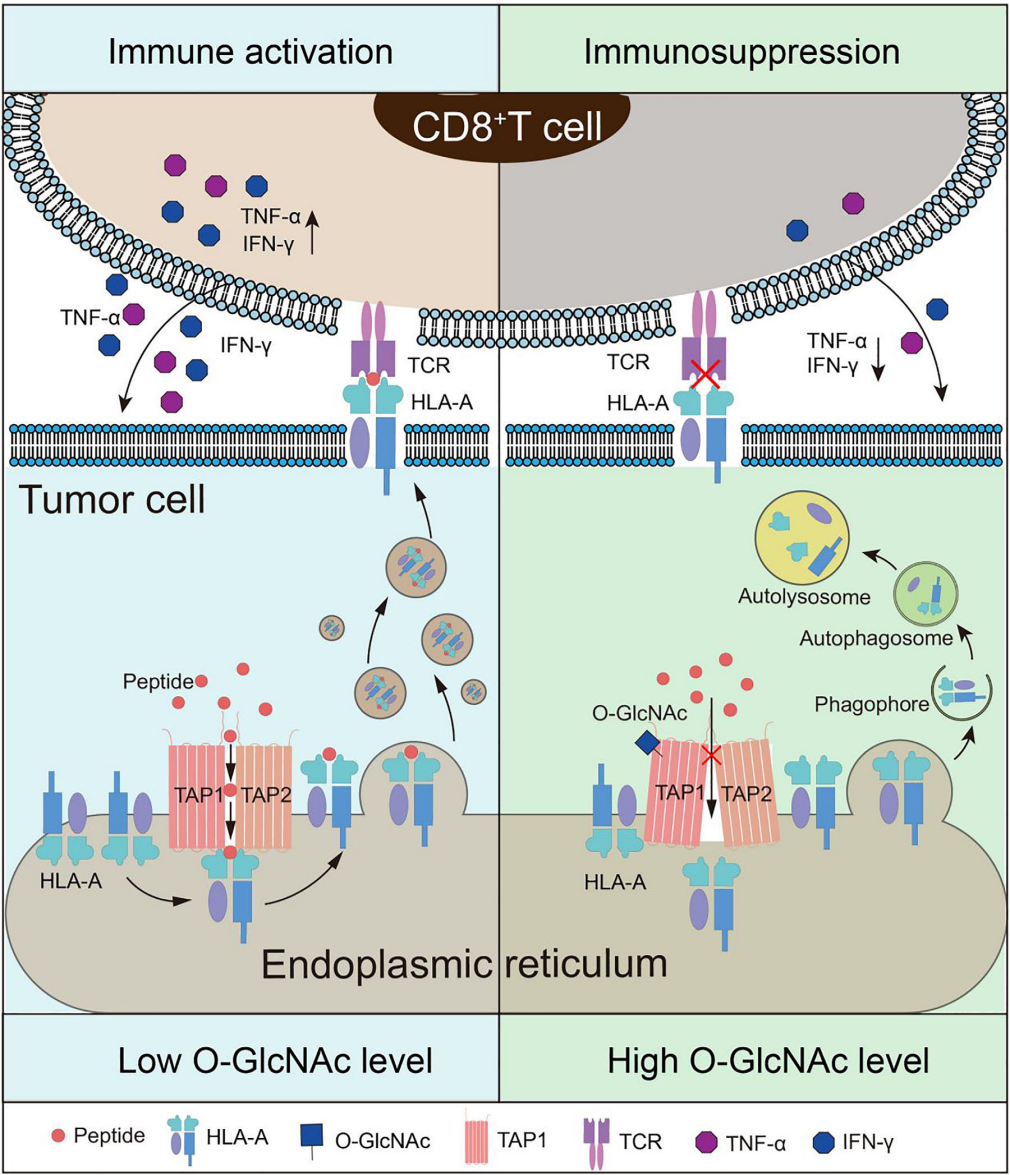
Schematic of O‐GlcNAcylation impact on antigen presentation via the TAP1/HLA‐A pathway.

In conclusion, this study demonstrated that O‐GlcNAc in TAP1 impairs its ability to transfer antigen peptides to HLA‐A. This consequently leads to HLA‐A degradation via the autophagy‐lysosomal pathway, thereby hindering CD8^+^ T cell recognition and killing of tumor cells, and ultimately facilitating immune evasion in bladder cancer (Figure 7).

## Author contributions

X.L. and F.G. conceived and devised the study. F.G., X.L., and J.W. designed experiments and analysis. J.W., X.R., L.W., Z.Z., L.L., Z.T., Z.J., Y.H., and Y.L. performed experiments. J.W. and X.R. performed bioinformatics and data statistical analyses. F.G. and X.L. supervised research, and wrote the manuscript in collaboration with J.W. All authors read and approved the finalized manuscript.

## Ethics Statement

With the approval of the Ethics Committee of Northwest University, we obtained human bladder cancer tissue and adjacent normal tissues from bladder cancer patients in the First Affiliated Hospital of Xi'an Jiaotong University. The study was approved by the Medical Ethics Committee and the Animal Care and Use Committee of Northwest University.

## Conflicts of Interest

The authors declare no conflicts of interest.

## Supporting information




**Supporting Information**: advs75365‐sup‐0001‐SuppMat.docx.

## Data Availability

The data that support the findings of this study are available in the supplementary material of this article.
