# Supplementary material for: O‐GlcNAcylated TAP1 Impairs Antigen Presentation and Promotes Immune Evasion in Bladder Cancer
Source: Adv Sci (Weinh). 2026 Apr 24:e19955. Online ahead of print. doi: 10.1002/advs.202519955 (PMC13334654; doi:10.1002/advs.202519955)
Supplement: Supplementary file 1 — Supporting Information: advs75365‐sup‐0001‐SuppMat.docx. [file ADVS-9999-e19955-s001.docx]

**Supplementary Information**

**Title:** **O-GlcNAc modification of TAP1 impairs antigen presentation and promotes immune evasion in bladder cancer**

Jinpeng Wu^1#^, Xueting Ren^2#^, Lu Wang^1^, Zhen Zhai^2^, Liang Liang^3^, Zengqi Tan^1,4^, Jiazhen Zhao^1^, Yuxuan Han^1^, Yuhan Li^5^, Feng Guan^1*^ and Xiang Li^1*^

^1^ Key Laboratory of Resource Biology and Biotechnology in Western China, Ministry of Education, Provincial Key Laboratory of Biotechnology, College of Life Sciences, Northwest University, Xi’an, Shaanxi, China

^2^ The Comprehensive Breast Care Center, The Second Affiliated Hospital of Xi'an Jiaotong University, Xi’an, Shaanxi, China

^3^ Department of Urology, the First Affiliated Hospital of Xi’an Jiaotong University, Xi’an, Shaanxi, China

^4^ Shaanxi Province Key Laboratory of Molecular Cardiology, School of Medicine, Northwest University, Xi’an, Shaanxi, China

^5^ Institute of Molecular and Translational Medicine (IMTM), and Department of Biochemistry and Molecular Biology, Xi'an Jiaotong University Health Science Center, Xi'an, Shaanxi, China

# These authors contributed equally to the study.

* Correspondence to: Feng Guan, guanfeng@nwu.edu.cn, or Xiang Li, xiangli@nwu.edu.cn. College of Life Science, Northwest University, 229 Taibai North Road, Xi’an, Shaanxi 710069, China.

**This PDF file includes:**

Supporting text

Figures S1 to S6

Tables S1 to S4

**Supplementary Materials and Mathods**

Western blotting

Cells were lysed in RIPA buffer (50 mmol/L Tris, pH 7.2, 1% Triton X-100, 0.5% sodium deoxycholate, 0.1% SDS, 150 mmol/L NaCl, 10 mmol/L MgCl_2_, and 5% glycerol) containing 1 μg/mL protease inhibitor cocktail. Proteins were separated by SDS-PAGE, transferred onto PVDF membranes (Bio-Rad, CA, USA), and blocked with 3% bovine serum albumin (Beyotime) in TBST buffer (20 mM Tris-HCl, 150 mM NaCl, 0.05% Tween 20, pH 8.0) for 1 h at 37℃. The PVDF membranes were incubated with primary antibodies overnight at 4℃, incubated with HRP-conjugated with secondary antibodies for 2 h at 37℃, and photographed with a gel documentation system (Tanon Science & Technology Co, Shanghai, China).

Flow cytometry

Cells were fixed with 4% paraformaldehyde and permeabilized at room temperature for 30 minutes. After that, cells were blocked in 3% BSA for an additional 30 minutes. Cells were incubated with a primary antibody for 1 h, incubated with a secondary antibody conjugated with Alexa Fluor 488, and subjected to FACS analysis (ACEA Biosciences; San Diego, CA, USA).

Enzyme‑linked immunosorbent assay (ELISA)

96-well ELISA plates (Jet Biofil, Guangdong, China) were coated with patient serum and incubated with shaking for 2 h at 37℃. The plates were then blocked with 3% BSA in PBS for 1 h at room temperature before being washed with PBS. The primary antibody was incubated overnight at 4℃, followed by washing with PBS, each for 5 minutes. Subsequently, the secondary antibody was incubated for 2 h at 37℃. Afterward, the TMB substrate kit was added, and the reaction was stopped by the addition of 2 M sulfuric acid. The optical density at 450 nm was determined using a plate reader.

Structural dynamics analysis

The crystal structures of TAP1 and HLA-A were taken from the UniProt database. The glycosylated form of TAP1 (TAP^O-GlcNAc^) and its site mutant (TAP1^S63A^) were modeled using AlphaFold. The TAP1–HLA-A complex structure was also predicted with AlphaFold, and the resulting models were subsequently used for root mean square deviation (RMSD) calculations in Discovery Studio.

**Supplementary Figures**

**
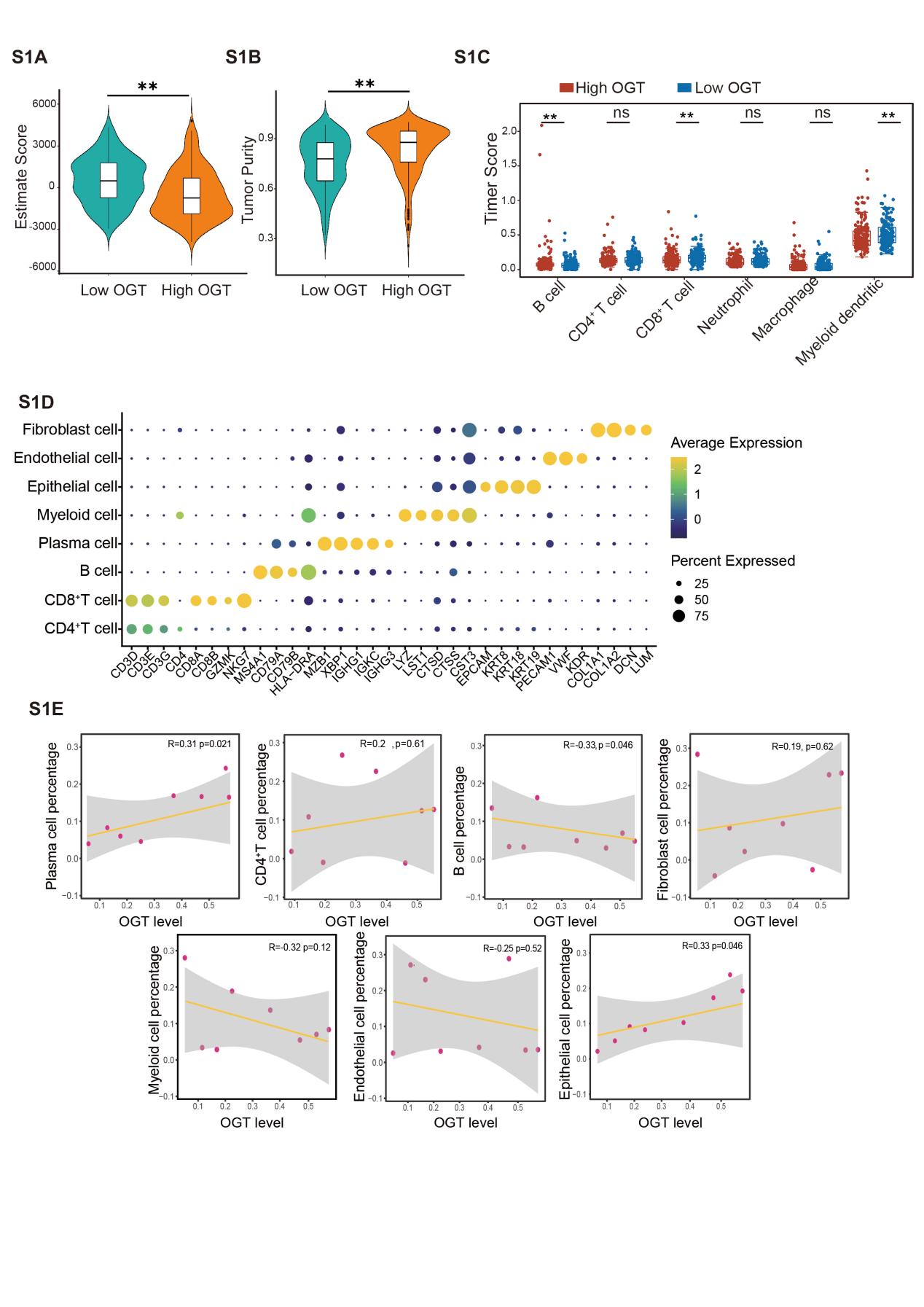
**

**Figure S1 A-B**. Estimate score (A) analysis and tumor purity (B) analysis of high and low OGT mRNA levels in bladder cancer patients (n=429). **C**. Timer score of high and low OGT expression in bladder cancer patients (n=429). **D**. Expression levels of cells markers. **E**.The correlation between OGT mRNA and CD8^+^T cell percentage. **E**. The correlation between OGT mRNA and CD4^+^T cell, plasma cell, B cell, fibroblast cell, myeloid cell, endothelial cell, epithelial cell percentage.


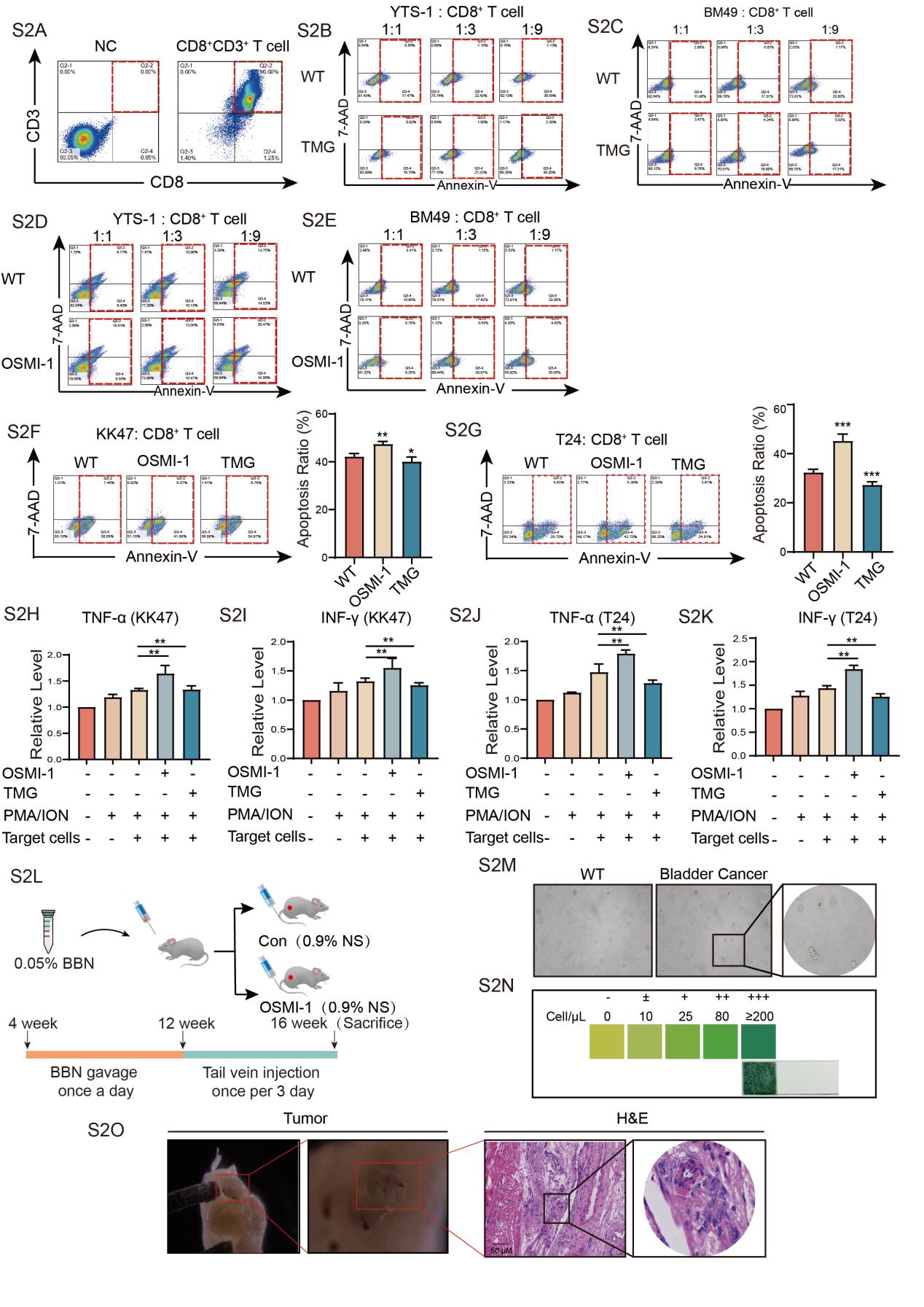


**Figure S2 A.** Identification of the CD8^+^ T cell isolated from human PBMCs by flow cytometry. **B-C**. CD8^+^ T cell-mediated cytotoxicity of YTS-1 cells (B) or MB49 cells (C) after treatment with 20 μM TMG for 48 h. **D-E**. CD8^+^ T cell-mediated cytotoxicity of YTS-1 cells (D) or MB49 cells (E) after treatment with 40 μg/mL OSMI-1 for 48 h. **F–G**. CD8^+^ T cell-mediated cytotoxicity of KK47 cells (F) or T24 cells (G) after treatment with 40 μg/mL OSMI-1 or 20 μM TMG for 48 h. **H–K**. TNF-α and IFN-γ levels in CD8^+^ T cells co-cultured with (H&I) KK47 or (J&K) T24 cells. **L**. Schematic of BBN induced mouse model of bladder cancer carcinogenesis. **M**. Hematuria was observed under the microscope. **N**. Hematuria was detected by hematuria indicator paper. **O.** H&E staining of bladder cancer tissue induced by BBN.


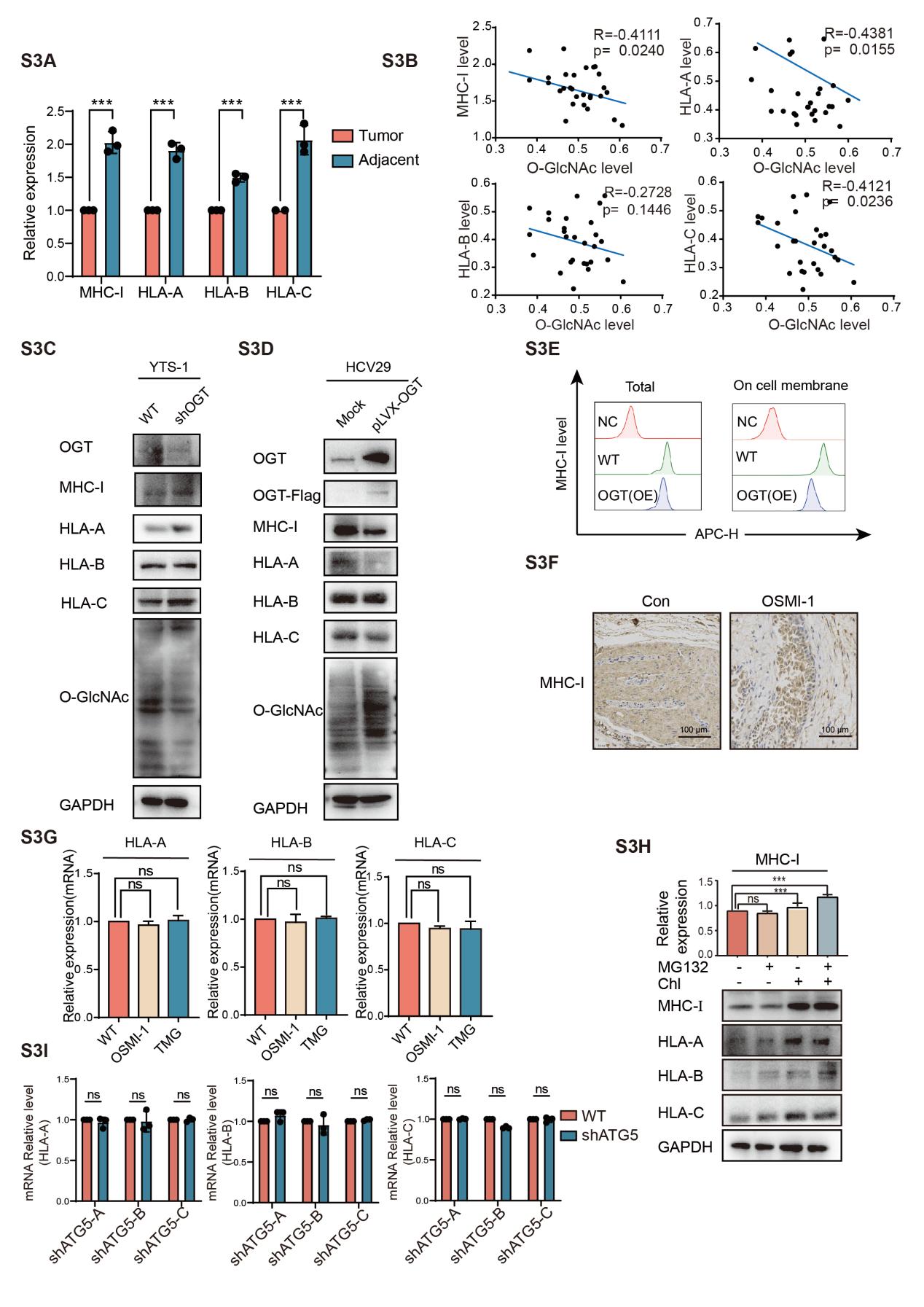


**Figure S3 A**. The Statistics of MHC-I, HLA-A, HLA-B and HLA-C in tumor and adjacent tissues. **B**. The Pearson correlation between O-GlcNAc and MHC-I, HLA-A, HLA-B, and HLA-C in bladder cancer tissues (n=30). **C-D**. Expression of OGT, Flag, MHC-I, HLA-A, HLA-B and HLA-C (C) in HCV29 cell line overexpressing OGT or (D) YTS-1 cell line silenced in OGT. **E**. Expression levels of the total of MHC-I and the cell surface level of MHC-I in the HCV29 cell line overexpressing OGT were detected by flow cytometry. **F**. IHC staining of MHC-I and HLA-A in paraffin sections of murine bladder cancer tissue. **G**. mRNA levels of HLA-A, HLA-B and HLA-C in YTS-1 cells treated with OSMI-1 or TMG. **H**. Level of MHC-I in YTS-1 cells treated with MG132 or Chl. **I**. Effect of *ATG5* knockdown on mRNA levels of HLA-A, HLA-B and HLA-C in YTS-1 cells.


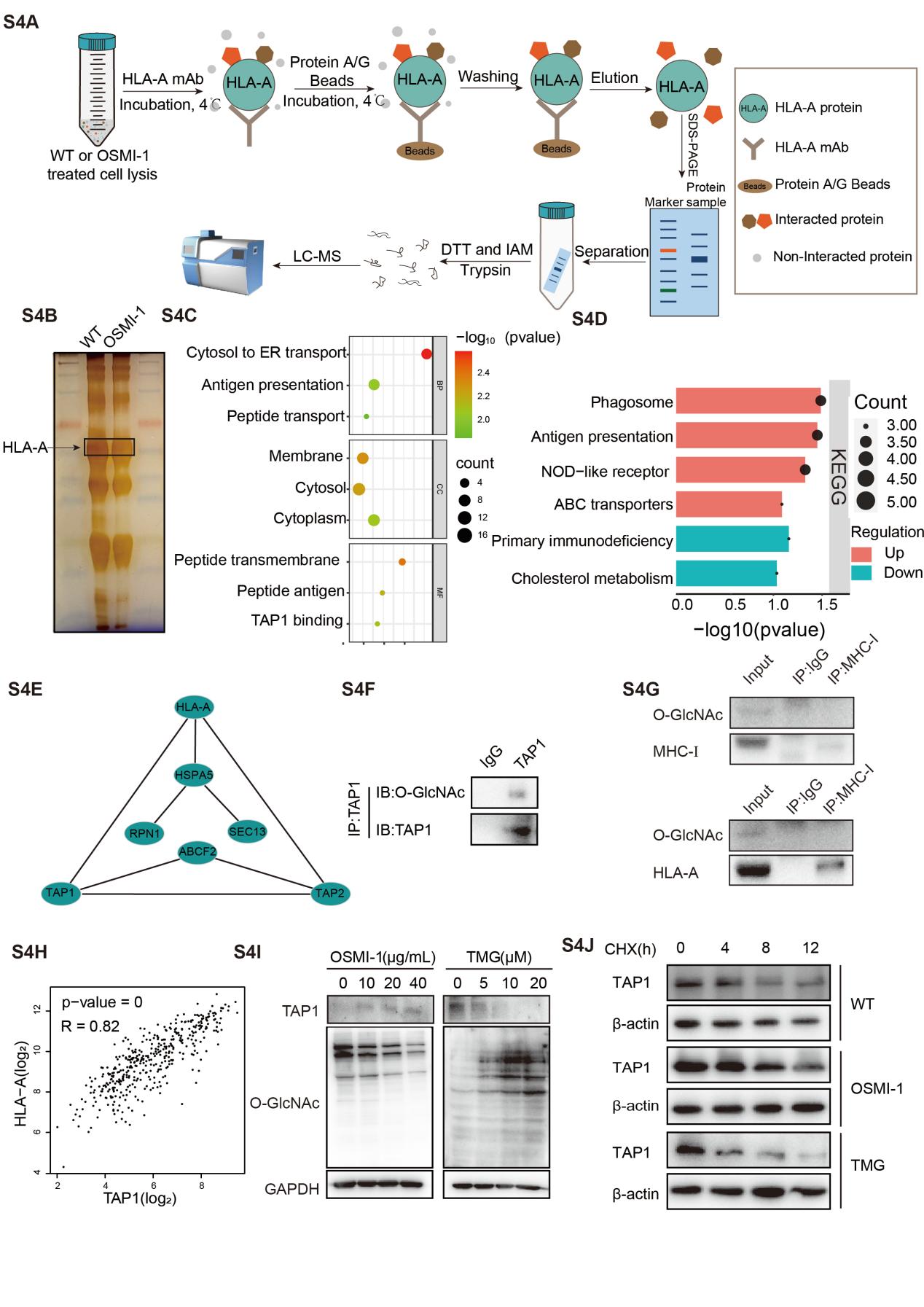


**Figure S4**

1. The Schematic of IP-MS. **B**. The quality of the IP-MS samples was monitored by silver staining. **C**. GO enrichment analysis of proteins interacting with HLA-A. **D**. KEGG analysis of the proteins interacting with HLA-A. **E**. Protein-protein interaction of proteins interacting with HLA-A, by Cytoscape software program. **F**. O-GlcNAcylation of TAP1 in YTS-1 cells were detected by Co-IP. **G**. O-GlcNAcylation of HLA-A and MHC-I in YTS-1 cells were detected by Co-IP. **H**. The correlation between TAP1 level and HLA-A level. **I**. Expression of TAP1, GAPDH and O-GlcNAc levels in YTS-1 cells treated with OSMI-1 or TMG were detected by western blot. **J**. The half- life of TAP1 in YTS- 1 treated with OSMI- 1 or TMG.


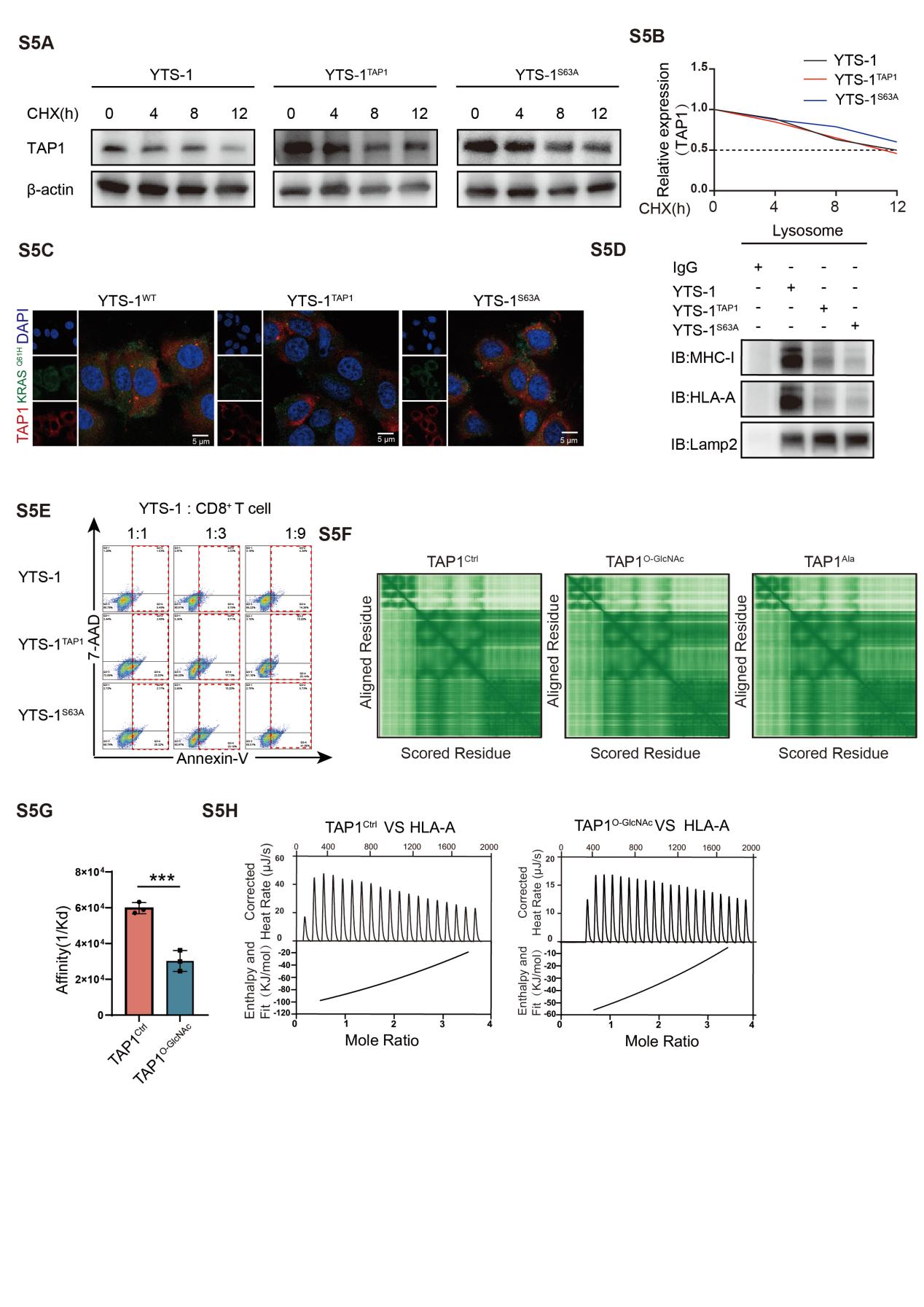


**Figure S5**

1. Expression of TAP1 in YTS-1^TAP1^ and YTS-1^Ser63A^ cell treated with CHX at various time points. **B**. The half-life of TAP1 in YTS-1^TAP1^ and YTS-1^Ser63A^ cell. **C**. Immunofluorescence staining analyzed the colocalization of TAP1/KRAS^Q61H^ in YTS-1, YTS-1^TAP1^ and YTS-1^S63A^ cells. **D**. Levels of MHC-I and HLA-A in lysosomes were detected in YTS-1, YTS-1^TAP1^, and YTS-1^Ser63^ cells. **E**. CD8^+^T cell-mediated cytotoxicity of YTS-1, YTS-1^TAP1^ and YTS-1^S63A^ was assayed by flow cytometry. **F**. The correlation of aligned residue and scored residue was detected in TAP1, TAP1^S63A^ and TAP1^O-GlcNAc^ model. **G**. Affinity between HLA-A and TAP1^Ctrl^ vs. TAP^O-GlcNAc^ was detected by ITC. **H**. The exothermic peaks from the ITC thermograms demonstrating the binding of TAP1^Ctrl^ and TAP1^O-GlcNAc^ to HLA-A are presented.


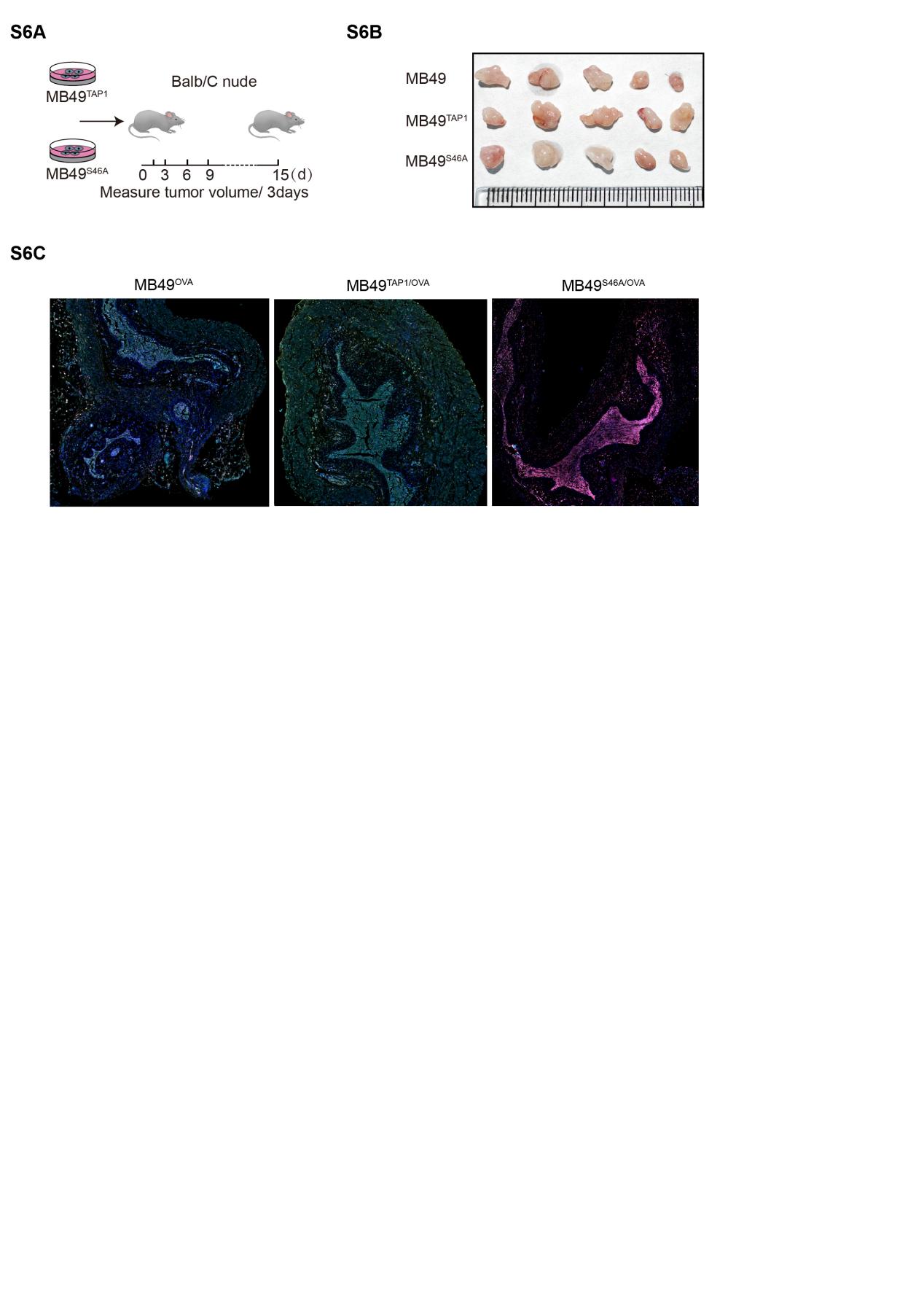


**Figure S6 A**. The schematic of xenograft mouse model. **B**. Photos of tumors from *BALB/c* nude mice injected with MB49, MB49^TAP1^, and MB49^S46A^ cells. **C**. Immunofluorescence staining of immune markers in tumor tissues.

**Supplementary Table S1**

| **Primer name** | **Target gene** | **Primer sequence (5’-3’)** |
| --- | --- | --- |
| β-actin-F | β-actin | CTCCATCCTGGCCTCGCTGT |
| β-actin-R |  | GCTGTCACCTTCACCGTTCC |
| ATG5-F | ATG5 | GACCCCGGTCCTCAAGGAA |
| ATG5-R |  | TGTAGCCCATTGCCATGTTGG |
| HLA-A-F | HLA-A | GATTACATCGCCTTGAACGAGG |
| HLA-A-R |  | AGAGACAGCGTGGTGAGTCAT |
| HLA-B-F | HLA-B | CAGTTCGTGAGGTTCGACAG |
| HLA-B-R |  | CAGCCGTACATGCTCTGGA |
| HLA-C-F | HLA-C | CCATGAGGTATTTGTGGACCG |
| HLA-C-R |  | TCTCGGACTCTCGTCGTCG |

**Supplementary Table S2**

| **Primer name** | **Target gene** | **Primer sequence (5’-3’)** |
| --- | --- | --- |
| TAP1-XhoI-F | TAP1 full-length | CCGCTCGAGATGGCTAGCTCTAGGTGTCCCGCT |
| TAP1-AgeI-R |  | ACCGGTTTCTGGAGCATCTGCAGGAGCCTGC |
| TAP1-S63-F | TAP1 Ser63 mutant | CTGGGCGGTGGGCCTGGCCCGCTGGGCCGTG |
| TAP1-S63-R |  | AGAGCACGGCCCAGCGGGCCAGGCCCACCG |
| ATG5-A-F | ATG5 | CCGGTCACCGCGGATGGGTAGATACATATCCTCGAGGATATGTATCTACCCATCCGC |
| ATG5-A-R |  | AATTCAAAAGCGGATGGGTAGATACATATCCTCGAGGATATGTATCTACCCATCCGC |
| ATG5-B-F | ATG5 | CCGGTCACCGGAAGAAGATGAAGATGAAGGCTCGAGCCTTCATCTTCATCTTCTTCC |
| ATG5-B-R |  | AATTCAAAAGGAAGAAGATGAAGATGAAGGCTCGAGCCTTCATCTTCATCTTCTTCC |
| ATG5-C-F | ATG5 | CCGGTCACCGCTGCAGATATGGAAGAATATCTCGAGATATTCTTCCATATCTGCAGC |
| ATG5-C-R |  | AATTCAAAAGCTGCAGATATGGAAGAATATCTCGAGATATTCTTCCATATCTGCAGC |
| shTAP1-F | TAP1 | AATTCAAAACGGGATCTATAACAACACCATTCCTCGAGGAATGGTGTTGTTATAGATCCCGTCTGCAGC |
| shTAP1-R |  | AATTCAAAAGGGATCTATAACAACACCATTCCTCGAGGAATGGTGTTGTTATAGATCCCGTGCAGC |

**Supplementary Table S3**

**The correlation between O-GlcNAc level and MHC-I, HLA-A/B/C.**

| Sample | O-GlcNAc | MHC-I | HLA-A | HLA-B | HLA-C |
| --- | --- | --- | --- | --- | --- |
| 1 | 0.381667 | 2.187 | 0.793667 | 0.418 | 0.479333 |
| 2 | 0.464667 | 2.21 | 0.594 | 0.429 | 0.392667 |
| 3 | 0.469667 | 1.227 | 0.601667 | 0.41 | 0.393 |
| 4 | 0.427667 | 1.816333 | 0.508667 | 0.472 | 0.456333 |
| 5 | 0.381667 | 1.784667 | 0.547333 | 0.513 | 0.457667 |
| 6 | 0.391 | 1.776 | 0.655667 | 0.344 | 0.474333 |
| 7 | 0.464667 | 1.671667 | 0.705667 | 0.44 | 0.549667 |
| 8 | 0.469667 | 1.860667 | 0.685 | 0.476 | 0.496333 |
| 9 | 0.427667 | 1.747 | 0.437333 | 0.496 | 0.375333 |
| 10 | 0.537 | 1.961667 | 0.498 | 0.374667 | 0.374667 |
| 11 | 0.487 | 1.458333 | 0.391 | 0.223 | 0.223 |
| 12 | 0.513667 | 1.445333 | 0.451333 | 0.387 | 0.387 |
| 13 | 0.540667 | 1.968333 | 0.515 | 0.416667 | 0.416667 |
| 14 | 0.480667 | 1.678 | 0.428667 | 0.319 | 0.319 |
| 15 | 0.55 | 1.867333 | 0.688667 | 0.531 | 0.531 |
| 16 | 0.495667 | 1.559333 | 0.744667 | 0.555667 | 0.555667 |
| 17 | 0.512333 | 1.584667 | 0.451333 | 0.313667 | 0.313667 |
| 18 | 0.455667 | 1.638667 | 0.437 | 0.279 | 0.279 |
| 19 | 0.554 | 1.567333 | 0.432333 | 0.393 | 0.359333 |
| 20 | 0.489667 | 1.851667 | 0.497667 | 0.408 | 0.283667 |
| 21 | 0.484667 | 1.663667 | 0.423333 | 0.326 | 0.287 |
| 22 | 0.518667 | 1.954667 | 0.466333 | 0.312 | 0.433333 |
| 23 | 0.528667 | 1.546333 | 0.439 | 0.319 | 0.412 |
| 24 | 0.529333 | 1.567667 | 0.404667 | 0.461 | 0.276667 |
| 25 | 0.562 | 1.680333 | 0.453667 | 0.557 | 0.336333 |
| 26 | 0.606333 | 1.168 | 0.475 | 0.248 | 0.248 |
| 27 | 0.522333 | 1.391667 | 0.426667 | 0.293 | 0.293 |
| 28 | 0.569333 | 1.616667 | 0.452667 | 0.327 | 0.327 |
| 29 | 0.587333 | 1.244 | 0.384 | 0.262667 | 0.262667 |
| 30 | 0.571667 | 1.611333 | 0.526333 | 0.365333 | 0.365333 |

**Supplementary Table S4**

**The correlation between TAP1 level and O-GlcNAc.**

| Sample | O-GlcNAc | TAP1 |
| --- | --- | --- |
| 1 | 2.120666667 | 1.734333333 |
| 2 | 2.086 | 1.263 |
| 3 | 1.876333333 | 2.417 |
| 4 | 1.859333333 | 1.541333333 |
| 5 | 1.747666667 | 1.631333333 |
| 6 | 1.686 | 2.025 |
| 7 | 1.623 | 1.804 |
| 8 | 1.511333333 | 1.515 |
| 9 | 1.417666667 | 3.605666667 |
| 10 | 1.403666667 | 1.186 |
| 11 | 1.365666667 | 2.274 |
| 12 | 1.346333333 | 2.200666667 |
| 13 | 1.32 | 2.424 |
| 14 | 1.293666667 | 1.534666667 |
| 15 | 1.291333333 | 2.171 |
| 16 | 1.218333333 | 2.037 |
| 17 | 1.195333333 | 2.369666667 |
| 18 | 1.148 | 2.393666667 |
| 19 | 1.059333333 | 3.369333333 |
| 20 | 1.024 | 3.111 |

**.**
